# New Treatment of Coryza

**Published:** 1855-07

**Authors:** 


New Treatment of Coryza.—In the Journal de Médecine et de Chirurgie Pratiques for November, 1854, M. Yvyonneau says that he has obtained great success in coryza by plugging up the external nares by introducing a plug of lint rolled in collodion. The organ, he says, is protected from the contact of cold air, and at the same time the disordered surface is bathed in warm air, and the moisture exhaled in respiration. The remedy has, in his hands, never failed to produce a cure in twenty-four hours.

